# Drafting injustice: overturning *Roe v. Wade*, spillover effects and reproductive rights in context

**DOI:** 10.1177/14647001221114611

**Published:** 2022-08-04

**Authors:** Candace Johnson

**Affiliations:** 3653 University of Guelph, Canada

 We all knew that this was coming. The United States Supreme Court decision that would overturn *Roe v. Wade* (1973). First, there was the *Planned Parenthood v. Casey* (1992) decision that allowed for abortion restrictions, provided that they did not constitute an ‘undue burden’ for the women seeking them. These restrictions include parental notification and consent laws, mandatory ultrasounds, waiting periods, counselling and an expanding array of Targeted Regulations of Abortion Providers (the TRAP laws), which made it difficult for many clinics to remain in operation. The *Casey* decision also replaced the trimester-based approach, rooted in a clear conception of stages of liberty and ‘State interest’, with the conception of ‘viability’, which allows, around the same amount of time for procuring an abortion (about two trimesters), yet, is a less concrete concept, more open to interpretation and contestation. The *Casey* decision, while nuanced and carefully considered, foreshadowed what was to come in (at least) two important ways: 1) by dismantling the *Roe v. Wade* framework, it ushered in the restrictions mentioned above; and 2) it was a 5–4 decision, indicating that the Court was on the edge of a full reversal of *Roe v. Wade*. With the judicial appointments during the Trump presidency – Neil Gorsuch, Brett Kavanaugh and Amy Coney Barrett – and the political nature of abortion and Supreme Court decision making, it was only a matter of waiting until a case came forward that clearly violated the viability standard of the existing constitutional protections.

That case was *Dobbs v. Jackson Women’s Health Organization* (2020), which concerned restrictions that did not permit abortion beyond fifteen weeks’ gestational age, despite the fact that ‘viability’ was at approximately twenty-four weeks. This was a clear violation of the Constitution, and therefore the appeal of the Mississippi case found its way to the US Supreme Court. The leaked draft decision, written by Justice Samuel Alito, surfaced on 2 May 2022 (Gerstein and Ward, 2022). And while the apparent decision was not surprising – it had been many years in the making, through court decisions, policy developments and strategic court appointments – it was nevertheless quite shocking. The language of the draft decision was a complete reversal of the decision in the *Roe v. Wade* case,^1^ and demonstrated the degree to which the American political system is polarised in ways that penalise women for trying to claim the autonomy and rights that are accessible and uncontested for their male counterparts.

The official decision in *Dobbs v. Jackson Women’s Health Organization* was handed down on 24 June 2022. This version of Alito’s majority opinion differed very little from the leaked draft, with the significant additions of critiques of the dissent by Justices Breyer, Sotomayor and Kagan, and of the concurring opinion of Chief Justice Roberts, who says he would ‘take a more measured course’, and that he concurs ‘only in the judgment’ (*Dobbs v. Jackson*, 2020: 1, 12). In other words, Chief Justice Roberts would have preferred not to discard the entire Roe/Casey framework, but rather to adjust (or discard) the viability standard. At the other extreme, the concurring opinion of Justice Thomas suggests that other rights should be reconsidered and possibly overturned: access to contraception, equal marriage and same-sex sexual activity among them.

In this *Interchange*, I would like to examine the ways in which reproductive rights are in need of a re-articulation, not just in the United States but in Canada and beyond. My argument is that this case – the fall of *Roe v. Wade* in the United States and its spillover effects – reveals the importance of defending what I call *reproductive rights in context*. This is the nexus between, or combined conceptual terrain of, reproductive rights *and* reproductive justice, rather than one or the other. Reproductive rights are often considered to be too narrow or acute, and not attentive enough to context or contingencies. However, the fall of *Roe* reminds that rights are inherently important; their legislative replacements, even if viable, are unlikely to be as effective.^2^ As Rosalind Petchesky explains, ‘control over one’s body is an essential part of being an individual with needs and rights, a concept that is the most powerful legacy of the liberal political tradition’ (1990: 4). However, she is careful to warn that assertions of rights are not enough, and further laments:

Feminists in the United States have not yet succeeded in communicating an alternative vision of women’s liberty, or their ‘personal/ bodily integrity,’ that moves beyond traditional liberal concepts of the private self. Such a vision requires a critical deconstruction of the class, race, and gender meanings that imbue current abortion discourse […] Those ingredients will not emerge from within the framework of the constitutional law (Petchesky, 1990: xxvi).

This lament suggests (but does not name) the broader approach of reproductive justice (Ross and Solinger, 2017; Bakhru, 2019), which offers additional lenses through which to consider reproductive decisions: race, sexuality, socio-economic status, history, positionality, ability, global/local location/context, human rights, among others. However, the sharpness of the rights claim can sometimes get lost within this (appropriately) broad approach. The emphasis on *rights* (to abortion) *in context* (regional and gender/racial) provides a lucid and strategic way to proceed, as it is focused on the powerful, political value of rights, yet it is committed to the project of reproductive justice. Such a focus is essential in analysing components of the *Dobbs* decision as well as the spillover effects of the decision, both discussed below.

## Rights reversal ground zero: the United States

If significant portions of Justice Alito’s majority opinion analyse the common law tradition, and the ways in which abortion is not ‘deeply rooted’ in the country’s ‘history and traditions’ and is inconsistent with commitments to ‘ordered liberty’, it also attends to what he calls ‘modern developments’ that should make abortion unnecessary for women. He mentions such transformations as improved societal attitudes that will protect ‘unmarried women’: workplace anti-discrimination policies, improved parental leave policies and medical insurance guarantees, as well as adoption ‘safe haven’ laws (*Dobbs v. Jackson*, 2020: 33). All of these ‘modern developments’, however, miss the point of the debate. Abortion was not a procedure available, as a result of a flawed judicial decision process, for shame-filled, mistake-ridden ‘unmarried women’. Abortion is a reproductive right that is for all women, all reproductive subjects.^3^ This is understood by many countries that provide safe and legal abortion and/or are in the process of liberalising abortion laws. The US is one of few that is moving in the opposite direction. This is a shocking reversal of reproductive rights and reproductive justice. Moreover, from a reproductive justice perspective, where are the ‘modern developments’ in the form of better health care access? More support for children with disabilities? Publicly funded contraception? (Beyond the coercive experiments with sterilisation and Norplant and Depo-Provera – see: Roberts, 1997: 104–149). Alito guesses at the racialised dimensions of the reproductive justice approach, but gets it wrong (*Dobbs v. Jackson*, 2020: 30). The problem is not simply that abortion is oversubscribed by Black women, but that the State provides too little of all other services. The overturning of *Roe* will disproportionately affect Black women in that they will be most likely to suffer from the inequality in access created by the patchwork of pro- and anti-choice states (Cai et al., 2022). States with trigger laws^4^ were positioned to immediately restrict abortion with limited exceptions, and included provisions for the criminalisation of providers. However, the spillover effects will potentially increase the criminalisation of abortion beyond the borders of the United States. This trend rejects the growing momentum towards calls for the abolition of the carceral state among feminists and instead increases state violence and the criminalisation of reproduction (see: Goodwin, 2020; Davis et al., 2022).

## Spillover effects: Canada and Latin America

The last time I was in Texas, in the border region, I crossed into Mexico at an entry point, travelling on foot. I was among hordes of people crossing over to buy pharmaceuticals, dentistry services, ceramics, *cacahuates* – cheaper, easier to acquire on the Mexican side of the border. American women are familiar with border crossings into Mexico, and medication abortion is widely available (De Guzman, 2022). Further, abortion is now decriminalised throughout all of Mexico, as a result of a Supreme Court ruling in 2021 (although not all states have yet liberalised their laws). Abortion is also decriminalised and accessible in Uruguay, Argentina, Colombia, Chile, Belize, Cuba and Guyana. Until 2012, abortion was only decriminalised and available^5^ in the latter three countries. With the overturn of *Roe*, the US defies the global trend towards the liberalisation of abortion laws, and increases the likelihood that it will exacerbate its problems in other areas as well, such as high maternal mortality rates with alarming racial disparities. The right to reproductive autonomy understood in a regional and global context can give clues about what ought to be done in a specific country or situation. Reproductive justice demands attention to the racialised dimensions, and the socio-economic dimensions, and the ableist dimensions, and the gendered dimensions, and the global dimensions of embodied experience and lived reality. This interacts with the right to abortion, but the two do not cancel each other out. Even if Alito’s ‘modern developments’ helped with embodied experience and lived reality for marginalised groups and individuals, we would still need, and be entitled to assert, a reproductive right to abortion.

Further, the reproductive justice framework reminds us to think about a community of others beyond our own borders. Loretta J. Ross and Rickie Solinger explain that, ‘we think of reproductive justice as an open source code that people have used to pursue fresh critical thinking regarding power and powerlessness’ (2017: 71), and that ‘reproductive justice argues that social institutions, the environment, economics, and culture affect each woman’s reproductive life. Reproductive justice activists invoke the global human rights system as the relevant legal framework using treaties [and] standards [while] moving beyond the U.S. Constitution’ (Asian Communities for Reproductive Justice, cited in Ross and Solinger, 2017: 69). As explained, it is important to consider the US in context as a way to appreciate the consequences of the *Dobbs* decision and its meaning in broad terms. It is also important to consider the spillover effects of the decision on countries like Canada and Guatemala, as two examples of countries that have deep connections to the US. Yet the impact of the reversal of *Roe* will be felt around the world, as US policies, politics and ideologies extend to many countries, and the end of constitutional protection for abortion in the US ‘would also likely have symbolic consequences globally, shaping the strategies and tactics of the transnational anti-abortion movement’ (Flowers, 2022).

A recent public opinion poll reveals that 46 per cent of Canadians think that the situation concerning abortion in the US will have an impact in Canada (Leger, 2022). We have already seen evidence of this, as some political parties have confirmed support for a woman’s right to choose, while others have asked members not to discuss abortion at all. Provincial governments have re-visited abortion policies, and pro-life actors are emboldened by the possibilities of a new jurisprudential hemispheric order. In more concrete and immediate terms, the concern is that pro-life groups will receive increased resources and encouragement from their American counterparts and that the status quo, which entails very limited abortion access in some parts of the country, will prevail or decline further. But perhaps of greater concern is the impact of the decision in countries like Guatemala. Jeff Abbott explains that some Latin American countries are making contentious and dangerous moves to the right, and that the fall of *Roe* will have an impact on women and the limited reproductive freedoms that they have. Quoting Ada Valenzuela, director of the National Union of Guatemalan Women, Abbott (2022) notes: ‘It is a bad message for the nations that are conditioned to [follow] U.S. policies […] It is a bad message about human rights. It [would be] a historical setback if this were to happen’. This demonstrates the importance of examining reproductive rights in context, and the significance and injustice of rights reversals in one location for women everywhere.

## Notes

1. A significant portion of the *Dobbs* decision focuses in detail on foetal protections and criminalisation of abortion that can be interpreted from the common law tradition. This is in complete opposition to Harry Blackmun’s (the Justice who wrote the majority opinion for the *Roe v. Wade* decision) opinion ‘that the criminalization of abortion was a relatively recent phenomenon, without roots in the English common-law tradition’ (Greenhouse, 2005: 92). Moreover, the examples provided by Alito in the majority opinion mainly focus on the sanctioning of abortion after quickening, which is around the point of ‘viability’ and consistent with both the *Roe* and *Casey* rulings. This is also discussed thoroughly in the *Roe* decision.

2. See McClain (2022) for a discussion of the codification of *Roe v. Wade*.

3. In this article I designate women as the subjects of abortion. While I refer here to women as a social category that also (mostly) maps onto a biological category, it is the case that transgender and non-binary individuals are subjects that seek out and access abortions. It is also important to recognise that women, as a social category, are the primary targets of gendered attacks by the State and other actors; the overturn of *Roe* is one of these attacks.

4. Thirteen states currently have trigger laws, although with the overturn of *Roe*, more states will likely restrict or ban abortion. For a review of trigger laws, see: Jiménez (2022).

5. Except in very restricted circumstances.
